# Case Report: Gallbladder perforation after endoscopic retrograde cholangiopancreatography—a rare complication

**DOI:** 10.3389/fmed.2026.1804101

**Published:** 2026-03-17

**Authors:** Ze-Ming Chen, Jing-Wen Xu, Wei-Feng Li, Xi-Qiu Yu, Qin-Hua Yang, Jin-Feng Wu

**Affiliations:** Department of Gastroenterology, Shenzhen Luohu People’s Hospital, Shenzhen, Guangdong, China

**Keywords:** case report, complication, endoscopic retrograde cholangiopancreatography, fully covered self-expanding metal stent, gallbladder perforation

## Abstract

**Background:**

Endoscopic retrograde cholangiopancreatography (ERCP) is a state-of-the-art diagnostic and therapeutic procedure for various pancreatic and biliary problems. In spite of the well-established safety of the procedure, there is still a risk of complications such as pancreatitis, cholangitis, bleeding and perforation. Gallbladder perforation has rarely been reported in association with ERCP.

**Case presentation:**

A 58-year-old male with pancreatic cancer was admitted for jaundice and underwent ERCP with fully covered self-expanding metal stent (FCSEMS) placement for obstructive jaundice, with significant postoperative bilirubin reduction. On the 10th postoperative day, the patient developed fever and severe right upper quadrant abdominal pain. Enhanced computed tomography (CT) of the upper abdomen confirmed gallbladder perforation complicated by perigallbladder abscess formation. Ultrasound-guided percutaneous abscess drainage and repeat ERCP with replacement of the common bile duct plastic stents ultimately resolved this complication, underscoring the critical importance of timely diagnosis and targeted intervention.

**Discussion:**

We report the first case of gallbladder perforation after ERCP in a patient with pancreatic cancer. Advanced imaging modalities, such as enhanced CT, play a crucial role in identifying ERCP complications. In this case, timely intervention through ultrasound-guided cholecystic abscess drainage and replacement with the plastic stents achieved successful resolution.

**Conclusion:**

This case underscores the rarity and clinical significance of ERCP-related gallbladder perforation, emphasizing the need for enhanced post-procedure scrutiny, especially in patients with relevant symptoms.

## Introduction

The primary complications of ERCP include pancreatitis, infection, hemorrhage, and duodenal perforation. Among these, acute pancreatitis is the most common, while duodenal perforation is the most severe (1). In contrast, gallbladder perforation following ERCP is an extremely rare complication (2), and reports of this adverse event in the literature are scarce. To our knowledge, this is the first case report documenting gallbladder perforation with perigallbladder abscess formation after FCSEMS placement during ERCP, which was successfully managed with ultrasound-guided percutaneous cholecystic abscess drainage and subsequent common bile duct plastic stents replacement.

## Case presentation

**Figure 1:**
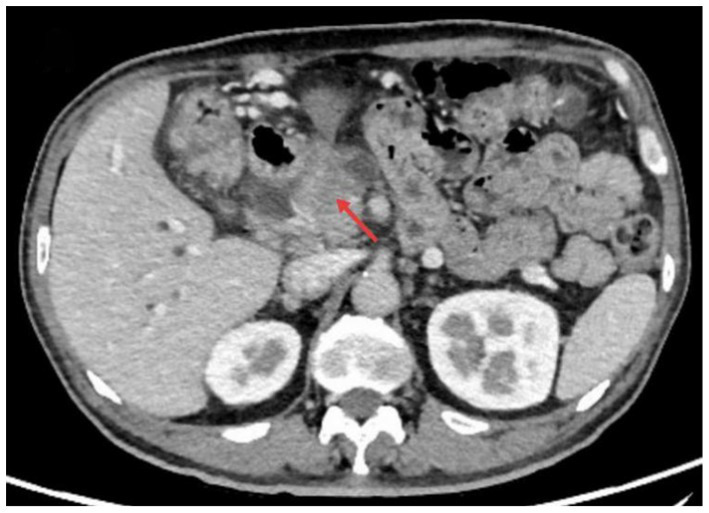
Pancreatic carcinoma compressing the distal common bile duct with resultant stenosis.

**Figure 2:**
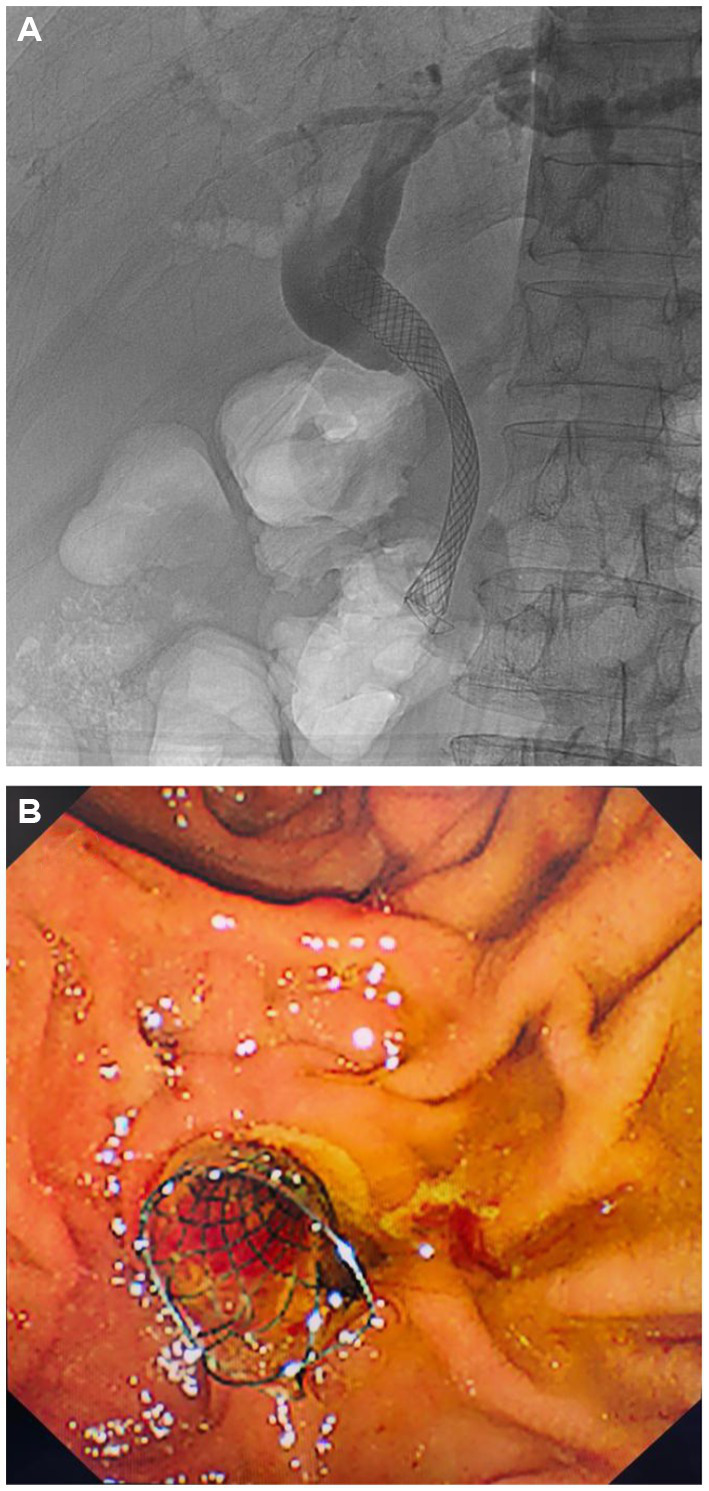
ERCP with placement of a FCSEMS. **(A)** Fluoroscopic view. **(B)** Endoscopic view.

A 58-year-old male with a history of pancreatic cancer was admitted to the hospital with jaundice for 5 days. He presented without abdominal pain or fever. Physical examination revealed icterus of the sclera and skin. The abdomen was non-tender with no rebound tenderness, and Murphy’s sign was negative. Laboratory tests showed a white blood cell count of 6.57 × 10^9/L, C-reactive protein (CRP) level of 5.67 mg/L, total bilirubin of 22.3 mg/dL (0–1.5 mg/dL), with direct bilirubinemia of 17.6 mg/dL (0–0.5 mg/dL), alanine aminotransferase 152u/L (9–50u/L), and aspartate aminotransferase 258u/L (15–40u/L). Enhanced CT of the upper abdomen revealed pancreatic cancer compressing the distal common bile duct, causing luminal narrowing (Figure 1). ERCP was performed, and a FCSEMS was placed based on the patient’s clinical condition (Figures 2A,B). On the night following ERCP, the patient experienced lower back pain, which was alleviated after applying a topical analgesic patch. Postoperatively, the patient’s bilirubin levels decreased significantly, and jaundice of the sclera and skin markedly improved.

**Figure 3:**
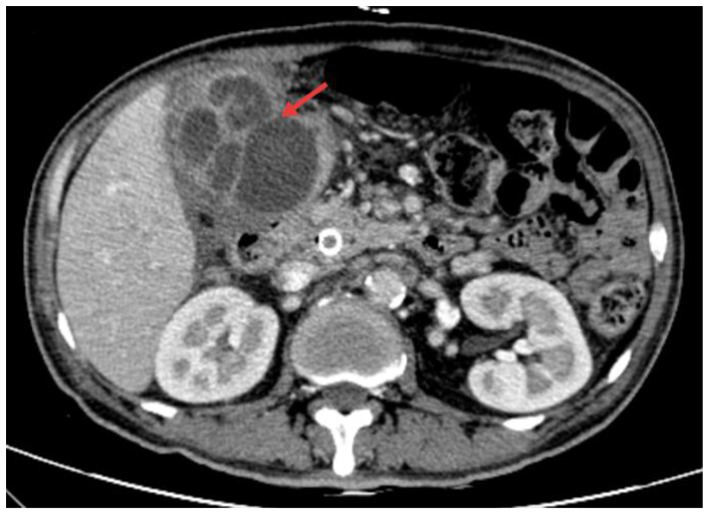
Gallbladder perforation complicated by perigallbladder abscess formation.

However, on the 10th day after ERCP, the patient developed a fever (39.0 °C) and was promptly treated with a third-generation cephalosporin combined with metronidazole. Subsequently, severe pain developed in the right upper abdomen. Physical examination revealed tenderness and rebound tenderness in the upper abdomen with positive Murphy’s sign. Laboratory findings showed a white blood cell count of 18.37 × 10^9/L, with neutrophils at 15.53 × 10^9/L, neutrophil ratio of 85.3%, a procalcitonin (PCT) level of 0.51 ng/mL, and a CRP level of 193.26 mg/L. Enhanced CT of the upper abdomen revealed gallbladder perforation with abscess formation (Figure 3).

**Figure 4:**
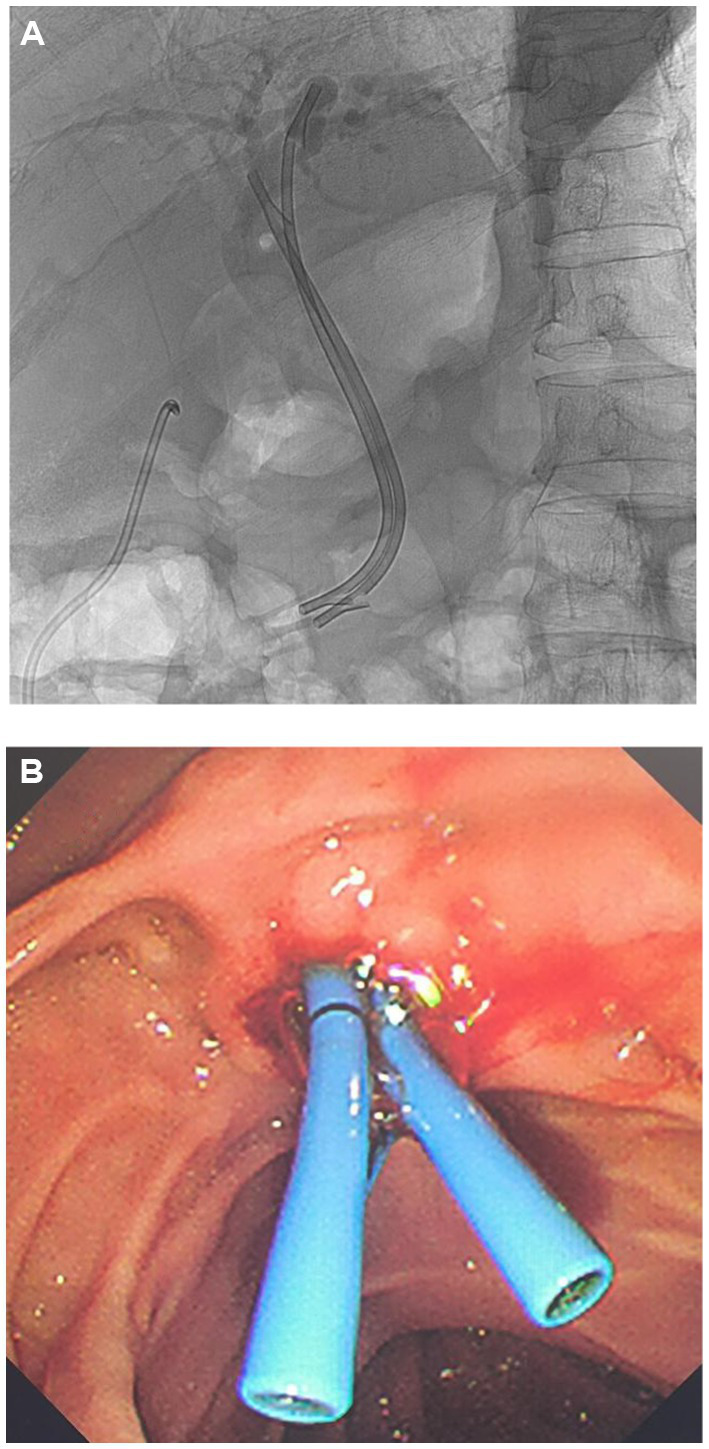
ERCP with placement of the common bile duct plastic stents. **(A)** Fluoroscopic view. **(B)** Endoscopic view.

**Figure 5:**
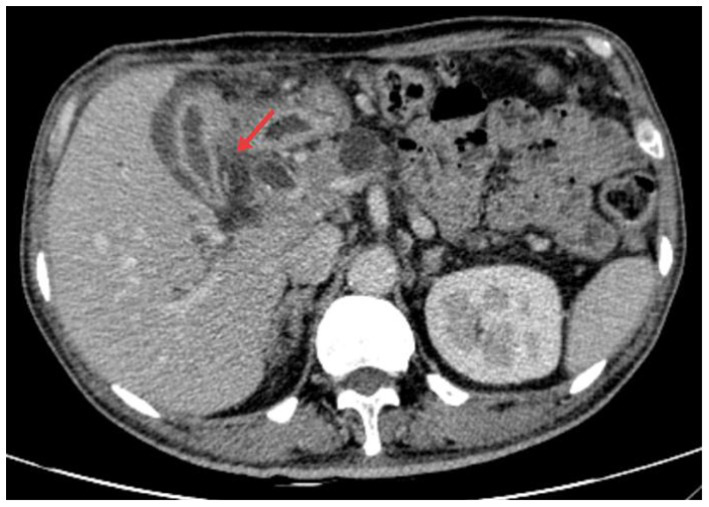
The gallbladder abscess has resolved.

Following diagnosis, the patient’s pancreatic cancer rendered emergency cholecystectomy a high-risk procedure. Consequently, the multidisciplinary team prioritized minimally invasive, organ-preserving surgical approaches to manage acute complications while avoiding excessive surgical trauma for a cancer patient with severe disease and poor prognosis. Therefore, we performed ultrasound-guided percutaneous drainage of the gallbladder abscess. Antibiotic susceptibility testing was conducted on the drained abscess fluid, and levofloxacin was continued according to the treatment plan. Subsequent ERCP was performed to replace the common bile duct plastic stents (Figures 4A,B). The patient’s temperature returned to normal, and right upper quadrant pain resolved. A subsequent enhanced CT scan of the upper abdomen showed resolution of the gallbladder-peritoneal abscess, with complete encapsulation of the gallbladder rupture site (Figure 5).

## Discussion

ERCP-related perforations are uncommon severe adverse events with an incidence between 0.1 and 1.5% (3). However, cases of gallbladder perforation following ERCP have not been previously reported. We know that FCSEMS are more prone to migration than uncovered self-expanding metal stents (UCSEMS), and migration into the cystic duct increases the risk of cholecystitis (4). In cases where the cystic duct inserts at a low-insertion (a congenital anatomical variation), the proximal stent may undergo slight displacement due to bile flow and intestinal peristalsis. This displacement can cause the proximal portion of the stent to obstruct the bile duct opening, leading to increased pressure within the gallbladder and subsequent rupture or perforation. In this case, we lack direct evidence linking stent-related effects (e.g., migration, mechanical irritation) to the perforation process. And based on evaluation of the patient’s preoperative enhanced CT of the upper abdomen and intraoperative ERCP cholangiography, we confirmed the absence of a low-insertion cystic duct. Since this is the first case report of gallbladder perforation after ERCP, the specific mechanism has not yet been fully elucidated. We consider the following reasons. First, the cystic duct may have been invaded by pancreatic cancer, resulting in narrowing. Furthermore, although we determined the appropriate length of the FCSEMS for this patient preoperatively, the FCSEMS expanded spontaneously and compressed the cystic duct after placement via ERCP. This increased pressure within the gallbladder, ultimately causing its rupture and perforation.

When lower back pain symptoms occur after ERCP stent placement, vigilance is warranted as it may indicate increased pressure in the cystic duct. Fever and right upper quadrant pain should raise suspicion for gallbladder perforation. Their clinical presentations can be misleading, leading to delayed diagnosis and management. Advanced imaging modalities, such as enhanced CT, play a crucial role in identifying ERCP complications. In this case, timely intervention through ultrasound-guided cholecystic abscess drainage and replacement with the plastic stents achieved successful resolution.

However, this case report has several limitations. Firstly, it is based on a single patient’s case, which limits the generalizability of the findings to a broader population. Secondly, since this is the first case report of gallbladder perforation after ERCP, the specific mechanism remains unclear. Third, current long-term follow-up data after patient discharge are limited, preventing comprehensive assessment of potential delayed complications such as recurrent bile duct strictures or chronic gallbladder-related sequelae. Lastly, due to the lack of comparable published cases of gallbladder perforation associated with ERCP, it is currently impossible to draw definitive conclusions regarding risk factors, optimal prevention strategies, and prognostic significance.

## Conclusion

This case highlights a rare but significant ERCP complication, advocating for enhanced scrutiny post-ERCP, especially if presenting lower back pain and right upper quadrant abdominal pain. Multidisciplinary approaches ensure comprehensive care, preventing delays in diagnosis and management of similar post-procedure complications. Promptly perform an enhanced CT scan of the upper abdomen. If gallbladder perforation is confirmed, promptly perform ultrasound-guided gallbladder abscess drainage and replace the stent with the plastic stents.

## Data Availability

The original contributions presented in the study are included in the article/supplementary material, further inquiries can be directed to the corresponding authors.
